# Hairy Root Transformation: A Useful Tool to Explore Gene Function and Expression in *Salix* spp. Recalcitrant to Transformation

**DOI:** 10.3389/fpls.2019.01427

**Published:** 2019-11-11

**Authors:** Carolina Gomes, Annabelle Dupas, Andrea Pagano, Jacqueline Grima-Pettenati, Jorge Almiro P. Paiva

**Affiliations:** ^1^Department of Integrative Plant Biology, Institute of Plant Genetics, Polish Academy of Sciences, Poznan, Poland; ^2^LRSV, Laboratoire de Recherche en Sciences Végétales, UPS, CNRS, Université Toulouse 3, Castanet Tolosan, France; ^3^Department of Biology and Biotechnology “L. Spallanzani”, University of Pavia, Pavia, Italy

**Keywords:** *Salix purpurea*, willow, domains rearranged methyltransferase 2 (DRM2), *Agrobacterium rhizogenes*-mediated transformation, pGWAY-0

## Abstract

Willow (*Salix* spp. L.) species are fast-growing trees and shrubs that have attracted emergent attention for their potential as feedstocks for bioenergy and biofuel production, as well as for pharmaceutical and phytoremediation applications. This economic and environmental potential has propelled the creation of several genetic and genomic resources for *Salix* spp. Furthermore, the recent availability of an annotated genome for *Salix purpurea* has pinpointed novel candidate genes underlying economically relevant traits. However, functional studies have been stalled by the lack of rapid and efficient coupled regeneration-transformation systems for *Salix purpurea* and *Salix* spp. in general. In this report, we describe a fast and highly efficient hairy root transformation protocol for *S. purpurea.* It was effective for different explant sources and *S. purpurea* genotypes, with efficiencies between 63.4% and 98.7%, and the screening of the transformed hairy roots was easily carried out using the fluorescent marker DsRed. To test the applicability of this hairy root transformation system for gene functional analysis, we transformed hairy roots with the vector pGWAY-*SpDRM2*, where the gene *SpDRM2* encoding a putative Domain Rearranged Methyltransferase (DRM) was placed under the control of the CaMV 35S constitutive promoter. Indeed, the transgenic hairy roots obtained exhibited significantly increased expression of *SpDRM2 as* compared to controls, demonstrating that this protocol is suitable for the medium/high-throughput functional characterization of candidate genes in *S. purpurea and* other recalcitrant *Salix* spp.

## Introduction

*Salix* spp. L. (willows) are very diverse, comprising more than 400 identified species spread over a wide variety of natural habitats (Sulima et al., 2018). Willows display a high morphological diversity, occurring in the growth forms of trees, shrubs, or subshrubs. Shrub willows (*Vetrix* sub-genus) are ideal biomass feedstocks for bioenergy and biofuel applications given the ease of vegetative propagation, fast growth in short-rotation coppices (SRC) and high biomass yields (Kuzovkina et al., 2008;Karp et al., 2011). Some *Salix* spp. can also be used in phytoremediation strategies as they are characterized by physiological adaptations and ecological resilience, rendering them particularly suitable for the clean-up of environmental contaminants (Kuzovkina and Quigley, 2005; Zalesny and Bauer, 2007). Besides, willow species such as *Salix purpurea* L. (purple willow) have great potential for the production of natural alternatives to synthetic aspirin, as their bark is a source of salicylic glycosides (SGs) (Sulima et al., 2017).

In the last few years, the development of genetic and genomics tools for *Salix* spp., together with extensive phenotyping efforts, have significantly extended our knowledge on the factors involved in trait determination and phenotypic adaptation in willows (Hanley and Karp, 2014; Sulima et al., 2017; Fabio and Smart, 2018; Sulima et al., 2018; Gouker et al., 2019). Furthermore, the recent availability of the *S. purpurea* genome (https://phytozome.jgi.doe.gov) coupled to transcriptomic studies (Carlson et al., 2017; Yanitch et al., 2017) allowed this species to become a model for the *Salix* genus. However, the functional characterization of new candidate genes has been hindered by the lack of rapid and efficient regeneration and transformation protocols for *S. purpurea* and *Salix* spp. in general.

*Salix* spp. are recalcitrant to both transformation and *in vitro* regeneration. There are few reports describing the *in vitro* regeneration of *Salix* plants (Grönroos et al., 1989; Stoehr et al., 1989; Lyyra et al., 2006), but only one reported the regeneration of a significant number of plantlets (Stoehr et al., 1989). Early attempts to regenerate transgenic willow species were also proven ineffective, as no shoots were regenerated from transformed *calli* (Vahala et al., 1989, Vahala et al.,1993). Yang et al. (2013) developed a coupled regeneration-transformation system for *Salix matsudana* Koidz using the embryo apical region of mature seeds as initial explant. Shoots were regenerated directly from cotyledonary nodes. The average transformation frequency was low, approximately 7%, and moreover, this method required a laborious screening of transformants, given the chimeric nature of transgenic plants produced. More recently, Guan et al. (2018) developed an *Agrobacterium tumefaciens*-mediated genetic transformation system using leaf-based *calli* of *S. mongolica* as explants for transformation. Differentiation of adventitious buds and rooting of plantlets was accomplished by adding different ratios of 2,4-dichlorophenoxyacetic acid (2,4-D), 6-benzyl aminopurine (BA), and naphthaleneacetic acid (NAA) into Murashige and Skoog (MS) medium. The *Agrobacterium*-mediated integration of the *β*-glucuronidase (*gus*) gene into *S. mongolica* genome of five transgenic lines was confirmed by Southern blot (Guan et al., 2018), but no data on transformation efficiency were provided.

*A. rhizogenes*-mediated hairy root transformation systems are particularly useful for species recalcitrant to transformation by *A. tumefaciens*, since much higher transformation efficiencies are obtained, and shorter transformation periods are attainable in comparison to *A. tumefaciens*-mediated transformation systems. Hairy root transformation systems have been previously established in woody species such as *Prunus* spp. (Bosselut et al., 2011), *Populus* spp. (Yoshida et al., 2015), *Eucalyptus grandis* (Plasencia et al., 2016), *Camelia sinensis* (Alagarsamy et al., 2018), and *Dryas* spp. (Billault-Penneteau et al., 2019). In poplar, hairy roots were used to study the role of the transcription factor *MYB182* on the regulation of proanthocyanidin and anthocyanin biosynthesis (Yoshida et al., 2015). In *Eucalyptus*, hairy roots were shown to be a suitable system for the functional characterization of genes involved in lignin biosynthesis, such as the *Eucalyptus cinnamoyl-CoA reductase1* (*EgCCR1*) (Plasencia et al., 2016). To our best knowledge, in *Salix* spp., the induction of hairy roots has only been described in *Salix alba* L. (Hauth and Beiderbeck, 1992) in a report dating back to the early nineties. However, the aim of this study was solely to improve root biomass by the production of *A. rhizogenes*-induced hairy roots, as the normal roots cultures of *Salix alba* presented slow growth rates. Given the nature of this study, no confirmation of putatively transformed hairy root lines was done. Therefore, there is still a need to develop a hairy root transformation protocol that would allow the rapid characterization of gene function in *Salix* spp.

**Figure 1:**
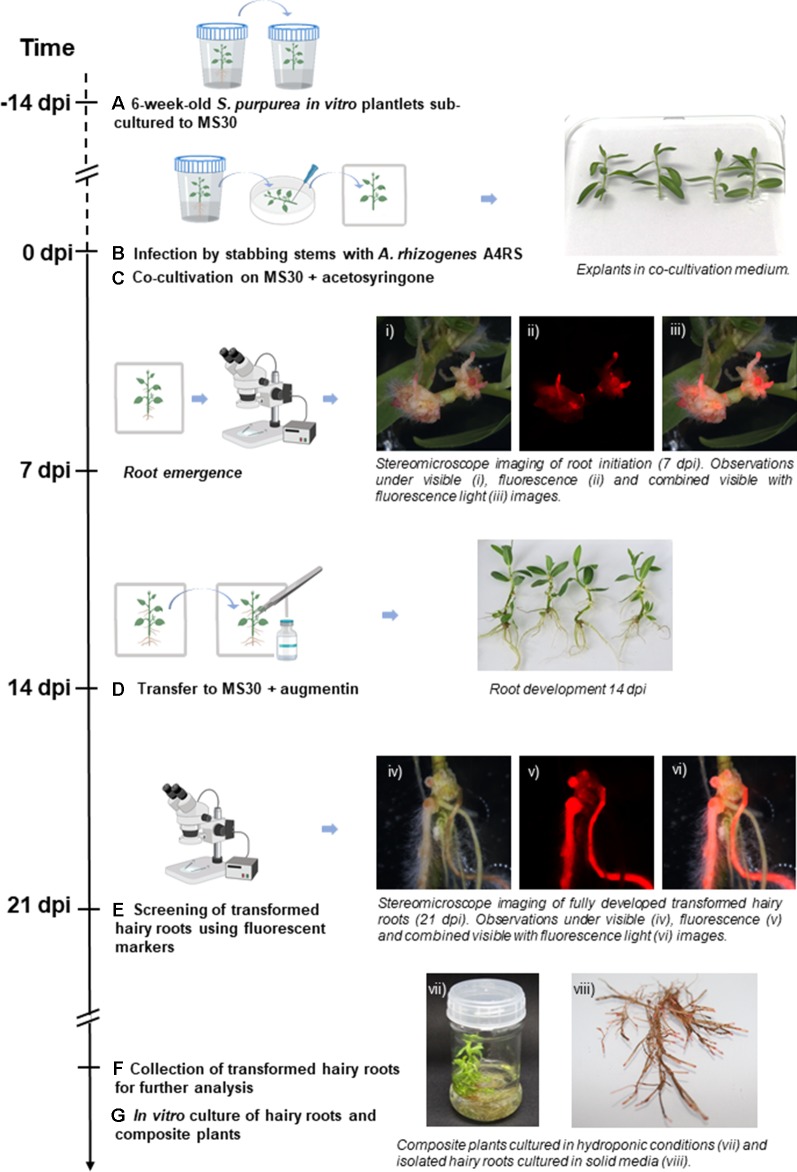
Workflow of the hairy root transformation of *Salix purpurea in vitro* plantlets by A4RS harboring a DsRed-based binary vector. Emerging DsRed fluorescent roots are already detected 7 days post inoculation (dpi). **(A)** 6-week-old *S. purpurea in vitro* plantlets are sub-cultured to MS medium with full strength macroelements and 30 g L^-1^ sucrose (MS30). **(B)** 14-day-old *S. purpurea in vitro* plantlets were infected by stabbing the stem with a needle swabbed with *Agrobacterium rhizogenes*. **(C)** Infected plants were co-cultivated with *A. rhizogenes* for 14 days on MS30 supplemented with acetosyringone under dim light. **(D)** Plants were transferred to MS30 medium supplemented with Augmentin. **(E)** Generated hairy roots were examined at 21 dpi under a stereo fluorescence microscope. **(F)** Co-transformed roots were excised and collected for further analysis. **(G)** *In vitro* culture of hairy roots and composite plants. Figure adapted from Plasencia et al. (2016), introducing the protocol specificities of hairy roots transformation for *S. purpurea*.

Here, we propose a reproducible, rapid and highly efficient *A. rhizogenes*-mediated hairy root transformation system for *S. purpurea* (**Figure 1**; details in **Supplementary Material S1**). In this method, the transformed hairy roots are detectable by fluorescent markers, allowing an easy and fast selection of transgenic roots. Our results suggest that this transformation system can potentially be applied to different genotypes, enabling gene functional studies in selected *S. purpurea* genotypes.

### Hairy Roots: A Highly Efficient Strategy for Purple Willow Transformation

Different hairy root transformation protocols were tested to check the effect of explant age, plant culture media, and genotype on the transformation efficiency. We initially tested four transformation protocols combining one genotype at two developmental stages and two culture media (**Supplementary Table S2.1**—Experiment A). DsRed fluorescence was easily detected in neo-formed *calli* at wounding sites and in emerging roots in average 7 days after inoculation (dpi), i.e. (**Figure 1**). The transformation efficiencies obtained 21 dpi were higher for two-week-old *S. purpurea* plantlets grown in MS30 (reaching 83.33%) with the other three tested conditions presenting lower efficiencies (between 63.41% and 67.86%). To validate the results of the first experiment and to check if the developed protocol could be applicable to different *S. purpurea* genotypes, a second transformation experiment (**Supplementary Table S2.1**—Experiment B) was performed comparing two non-related genotypes, ELB3/6 and ELB2/5, using two-week-old *in vitro* clonal lines of *S. purpurea* grown in MS30, as this was shown to be the best condition to maximize the transformation efficiency. In this second experiment, the transformation efficiencies were slightly higher in both genotypes (above 86%), showing that the developed protocol is reproducible, rapid, and highly efficient (**Supplementary Table S2.1**—Experiment B). Moreover, we observed that isolated transformed hairy roots were able to continue to grow in solid media, and composite plants could be grown in hydroponic culture, allowing the maintenance and multiplication of composite plants and hairy roots for further experiments.

### Application of Hairy Roots Transformation of *S. purpurea* for Gene Functional Analysis

**Figure 2:**
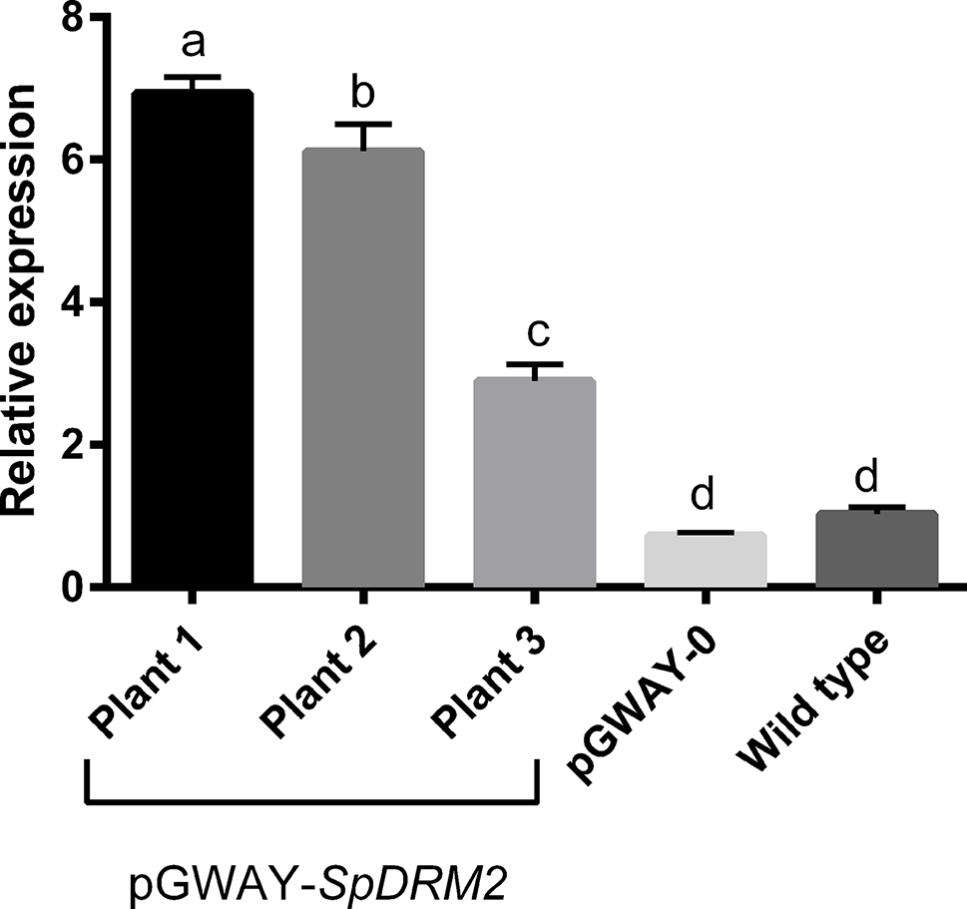
Relative expression of SpDRM2 in transformed pGWAY-SpDRM2 hairy roots collected in three composite plants, in hairy roots transformed with pGWAY-0 (empty vectors) and wild-type roots. Relative expression is expressed in arbitrary units, by comparing with the expression in wild-type roots. Bars indicate the average of relative expression for pGWAY-DRM2 hairy roots (n = 3; technical replicates) and for pGWAY—0 hairy roots or wild type roots (n = 9 3 biological × 3 technical replicates). Standard error of mean is also indicated above the bars. Different letters above the bars indicate significant differences between conditions (Tukey’s comparison test P-value < 0.05).

To confirm that this transformation approach was suitable for functional gene characterization and gene function hypothesis-testing, we transformed roots with *SpDRM2* (SapurV1A.0571s0130), a gene encoding a putative DNA methyltransferase with rearranged catalytic domains (For details on cloning of *SpDRM2* see **Supplementary Data S1**, **Supplementary Table S2.2**, and **Supplementary Figure S1**). This gene is an ortholog of *DRM2* of *Arabidopsis thaliana* (AT5G14620), where it was shown to encode an enzyme involved in *de novo* DNA methylation and gene silencing (Cao and Jacobsen, 2002). Transgenic hairy roots potentially containing the construct pGWAY-*SpDRM2* were screened using DsRed and transformation efficiencies were comparable to those obtained with pGWAY-0 (98.75% for 82 analyzed plants). Gene expression analysis by RT-qPCR confirmed the overexpression of *SpDRM2* in pools of hairy roots transformed with pGWAY-*SpDRM2*, collected from three different composite plants (**Figure 2**) (**Supplementary Data S1**, and **Supplementary Table S.2.3**). Comparing wild-type roots to roots transformed with pGWAY-0 (empty vector), no significant difference was observed in the transcript level of *SpDRM2*, indicating that the endogenous gene expression was not affected by the transformation process. In contrast, the expression level was significantly increased by 2.9-fold to 6.9-fold in *SpDRM2*-overexpressing lines as compared to wild-type (For details on cloning of SpDRM2 and RT-qPCR analysis of transformed hairy roots check S1- Detailed Material and methods, **Supplementary Table S2.2** and **Supplementary Table S2.3**). Based on the results obtained, we expect that this transformation method will be a valuable tool for the medium-/high-throughput functional characterization of candidate genes in *S. purpurea*.

## Discussion

Current *A. tumefaciens*-mediated transformation procedures of willows (Vahala et al., 1989; Yang et al., 2013; Guan et al., 2018) still limit functional studies in *Salix* spp. As they are laborious, time-consuming, genotype-dependent, and hindered by low transformation efficiencies. Furthermore, no stable transformation protocol is currently available for *S. viminalis* and *S. purpurea*, two relevant willow species for the production of biofuel and pharmaceutical compounds, respectively. Thus, the use of *A. rhizogenes* to induce transformed hairy roots represents a novel approach in *Salix*, as these species are recalcitrant to regeneration and transformation with *A. tumefaciens*. Previous reports suggest that hairy roots can be induced on a wide range of woody species, using *in vitro* (Alpizar et al., 2006; Bosselut et al., 2011; Yoshida et al., 2015; Plasencia et al., 2016) and *in planta* strategies (Alagarsamy et al., 2018), and from different explant types, e.g. seedlings, roots, stems, and leaves. In the present study, *A. rhizogenes* strain A4RS harboring the vector PGWAY-0 (Plasencia-Casavedall, 2015; Plasencia et al., 2016) was proven highly effective at inducing transgenic hairy roots in *S. purpurea* in explants with different ages and different genotypes. Transgenic hairy roots were easily detectable using the fluorescent marker DsRed, with emerging transformed roots appearing in average 7 days after *A. rhizogenes* inoculation. The fluorescent marker DsRed facilitates the identification of transgenic roots (which can be selected in the absence or in addition to antibiotic selection), allowing the non-destructive and precocious identification of transgenic roots (Limpens et al, 2004; Chabaud et al., 2006; Meng et al., 2019). High efficiency of hairy root transformation (ranging from 63% to 98%) was achieved for all tested conditions. The hairy root transformation efficiencies obtained in this report are similar to those reported for other woody species (Alpizar et al., 2006; Bosselut et al., 2011; Plasencia et al., 2016; Alagarsamy et al., 2018), legumes (Estrada-Navarrete et al., 2007; Aggarwal et al., 2018), and tomato (Ho-Plágaro et al., 2018). However, most available protocols use seedlings as explant for hairy root induction while in this report we used *in vitro*-cultured plantlets of *S. purpurea* as explants. The use of *in vitro*-cultured clonal lines has the advantage of not requiring the sterilization and germination of seeds prior to transformation, which can be limiting steps in the protocol in case of seed contamination, poor seed germination, and limited seedling growth (Alagarsamy et al., 2018). Contrastingly, *in vitro*-cultured clonal lines only require simple maintenance procedures and can be an almost continuously available source of explants for hairy root induction experiments. Besides, the use of clonal lines allows testing the expression of target genes in a plant material with identical genetic background.

Using the developed protocol (**Figure 1**, and **Supplementary Material S1**) we were able to achieve very high hairy root co-transformation efficiencies for both tested *S. purpurea* genotypes, suggesting that this hairy root transformation protocol can potentially be transferred to other *S. purpurea* genotypes. Nonetheless, more experiments should be done to test the susceptibility to *A. rhizogenes* in different *S. purpurea* genotypes and other willows species. A main limitation of this protocol is that it does not allow the transformation of above ground plant tissues other than hairy roots. Still, this can also represent an advantage when investigating root-shoot interactions in composite plant systems, to study, for example, the transport of small regulatory and signaling molecules (e.g. sRNAs, peptides and metabolites) between roots and aerial components of the plant. Transgenic hairy roots and composite plant systems can be used to study resistance against different biotic (Mellor et al., 2012; Xue et al., 2017) and abiotic stresses (Kajikawa et al., 2010; An et al., 2017; Du et al., 2018; Li et al., 2019), mycorrhizal associations, and root symbioses (Clemow et al., 2011; Indrasumunar et al., 2015;Billault-Penneteau et al., 2019). Furthermore, the use of the CRISPR/Cas9 technology with hairy root transformation has emerged as an efficient tool for plant genome editing and gene functional studies (Ron et al., 2014; Wang et al., 2016;Du et al., 2018), thus opening a whole new range of applications for *Salix* spp., such as the manipulation of target biosynthetic pathways through multiplexed genome editing. Indeed, as each single hairy root represents a single transformation event and can continue to grow autonomously, the system can be particularly useful as a medium-/high-throughput tool for functional analysis and biotechnological applications. The overexpression of *SpDRM2* in hairy roots transformed with the pGWAY-*SpDRM2* construct demonstrated the potential of *S. purpurea* hairy root transformation as a homologous, versatile, and efficient system that will enable the rapid validation of candidate genes and gene mining in *S. purpurea* and other recalcitrant willow species.

## Data Availability Statement

The datasets generated for this study are available on request to the corresponding author.

## Author Contributions

JG-P and JP conceived the study and designed the experiments. CG, AD, AP, and JP performed the experiments and analysed the results. CG, AP, JG-P, and JP wrote the manuscript. All authors read and approved the final manuscript.

## Funding

This work was supported NCN project Sonata-bis UMO-2015/18/E/NZ2/00694 (PurpleWalls Project). CG acknowledges a PhD scholarship in the frame of PurpleWalls Project and the Short-Term Scientific Mission (STSM) grant in the frame of COST Action 15223. AP acknowledges the Erasmus+ program of the European Union and the IUSS (University School for Advanced Studies) of Pavia. JP acknowledges his research contract in the frame of EU-FP7-ERAChairs-PilotCAll-2013 project “Biotalent—The creation of the Department of Integrative Plant Biology” (FP7-REGPOT-621321) and the Polish financial sources for education in the years 2015–2019 allocated to an international co-financed project.

## Conflict of Interest

The authors declare that the research was conducted in the absence of any commercial or financial relationships that could be considered a potential conflict of interest.
