# Supplementary material for: Hairy Root Transformation: A Useful Tool to Explore Gene Function and Expression in Salix spp. Recalcitrant to Transformation
Source: Front Plant Sci. 2019 Nov 11;10:1427. doi: 10.3389/fpls.2019.01427 (PMC6859806; doi:10.3389/fpls.2019.01427)
Supplement: Supplementary file 1 [file DataSheet_1.pdf]

Supplementary Material

S1- Detailed Material and methods

Plant material

Salix purpurea in vitro propagated plantlets were obtained from bud culture. Buds were collected on purple willow stems of the PSP43 and PSP33 genotypes of the *S. purpurea* germplasm collection at the University Warmia Mazuria in Olsztyn (Poland) (Sulima et al., 2017a, 2017b). Actively growing stems of approximately 5-10 mm in diameter were cut into 0.5-1 cm nodal segments containing one or two axillary buds and surface-sterilized for 15 min in a 10% (v/v) sodium hypochlorite solution. After three rinses with sterile water, the stem sections were put in upright position in plates containing MS30 - full strength Murashige and Skoog (MS medium, Sigma-Aldrich, St. Louis, USA), 30 g L^{-1} sucrose (Duchefa, The Netherlands) and solidified with 8 g L^{-1} agar (Micro-agar, Duchefa, The Netherlands). The plants were kept in controlled conditions (20 µmol m^{-2} s^{-1} applied as cool white fluorescent light, 16 h light/8 h dark photoperiod, 40% humidity, day/night temperature of 20-22 °C). After, 3-4 weeks, 1-1.5 cm plantlets were excised from the stem and sub-cultured to fresh MS30. To maintain and propagate the *S. purpurea in vitro* culture, 1.5-2 cm explants (with two axillary buds) were sub-cultured to fresh MS30 every 6 weeks.

Agrobacterium rhizogenes strains and culture media

The *Agrobacterium rhizogenes* strain A4RS (Jouanin et al., 1986) was used in all hairy-root induction experiments. The binary vectors were introduced into *A. rhizogenes* A4RS strain by electroporation (Nagel et al., 1990) and transformants were selected on 50 mg L^{-1} kanamycin, 50 mg L^{-1} rifampicin and 100 mg L^{-1} spectinomycin. Prior to infection transformed A4RS strains were grown for 72 h at 28 °C in AG solid media (Franche et al., 1997) supplemented with 100 µM acetosyringone (Sigma-

Aldrich, St. Louis, USA), 50 mg L^{-1} kanamycin (Duchefa, The Netherlands), 50 mg L^{-1} rifampicin (Sigma-Aldrich, St. Louis, USA) and 100 mg L^{-1} spectinomycin (Sigma-Aldrich, St. Louis, USA).

Construction of plant expression vectors

cDNA was reverse transcribed from 1 µg of total RNA samples isolated from buds of field-grown *S. purpurea*. Reverse transcription was carried out using the SuperScript™ IV First-Strand Synthesis System (Invitrogen, Carlsbad, CA, USA) and random primers, according to the manufacturer's instructions. The complete coding sequence of *SpDRM2* (SapurV1A.0571s0130.1) was amplified using template-specific primers containing 12 nucleotides of the Gateway attB sites (primers DRM2_ins_cds_F and DRM2_ins_cds_R, Supplementary Table S2.2) and universal attB adapter primers (primers attB1_adapter and attB2_adapter, Supplementary Table S2.2) in a 2-step PCR protocol developed by Invitrogen. The attB-PCR products were gel purified using the Zymoclean Gel DNA Recovery Kit (Zymo Research, Irvin CA, USA), and cloned into the Gateway entry vector pENTR221 (Invitrogen, Carlsbad, CA, USA) using BP clonase II (Invitrogen, Carlsbad, CA, USA) to obtain the construct pENTR_SpDRM2. Selected entry clones were mobilized to the Gateway-compatible plant expression vector pGWAY-0 using the LR clonase II (Invitrogen, Carlsbad, CA, USA) to obtain the expression construct pGWAY-*SpDRM2*. Entry and expression clones were screened by colony PCR and validated by Sanger sequencing.

Induction of hairy roots and screening of transformed hairy roots

A stem-stabbing infection protocol that has already been proven efficient in the induction of hairy roots in other woody species (Plasencia et al., 2016) was used in all hairy root transformation experiments. Briefly, an *Agrobacterium*-infected 0.45 mm-thick needle was used to inoculate the *S. purpurea in vitro* plantlets in four different sites. To test the effect of explant age on the induction of hairy roots,

two types of plant material were used: two-weeks-old and five-weeks-old *in vitro* grown PSP43 plantlets. After inoculation, the explants were placed in Petri dishes containing two different types of media: MS 1/2 macro (1/2 strength of macroelements, 30 g L^{-1} of sucrose and 8 g L^{-1} agar) and MS30, both supplemented with 100 µM acetosyringone (Duchefa, The Netherlands). To test the effect of genotype on the induction of hairy roots, two types of plant material were used: two-week-old *in vitro* grown PSP43 and PSP33 plantlets, respectively. After inoculation, the explants were placed in Petri dishes containing MS30 supplemented with 100 µM acetosyringone (Duchefa, The Netherlands). In all transformation experiments, at least 30 plants were tested for each condition. Inoculated explants were kept in co-culture with *Agrobacterium* for 14 days at 20-22 °C, 40% humidity and under dim light (7 µmol m^{-2} s^{-1}, 8- to 16-h photoperiod conditions). After 14 days, plants were transferred to the MS 1/2 macro or MS30 culture media without acetosyringone and supplemented with 300 mg L^{-1} augmentin (amoxicillin/clavulanic acid) to prevent the growth of *Agrobacterium* (Ieamkhang and Chatchawankanphanich, 2005). This step was done every 14 days to keep the antibiotic selection. Composite plants were grown in controlled conditions (20 µmol m^{-2} s^{-1}, 8- to 16-h photoperiod, 40% humidity, day/night temperature of 20-22 °C). The screening of co-transformed hairy roots was performed 21 days after infection using an automated stereomicroscope Leica M205 FA equipped with THUNDER Model Organism Imager (Leica Microsystems GmbH, Wetzlar, Germany) to detect DsRed fluorescence. Transformed hairy roots were isolated for further analysis.

RNA extraction and reverse transcription quantitative polymerase chain reaction (RT-qPCR)

One-month-old transformed hairy roots and wild-type roots were collected from three composite plants. Total RNA was extracted using the Spectrum Plant Total RNA Kit (Sigma-Aldrich, Inc., Germany) according to the manufacturer's instructions. The integrity of the RNA samples was visually verified by agarose gel electrophoresis. Additionally, the quality and quantity of extracted total RNA

was determined using NanoDrop™ One Microvolume UV-Vis Spectrophotometer (ThermoScientific, Waltham, MA, USA) and Qubit™ RNA BR Assay Kit (Invitrogen, Carlsbad, CA, USA). The isolated RNA samples were used for further RT-qPCR analysis. The first-strand cDNA was synthesized in biological triplicates from 140 ng total RNA aliquots using the GoScript™ Reverse Transcription System (Promega, Madison, WI, USA) following the kit instructions. Transcript abundances were quantified by RT-qPCR using the SensiFAST™ SYBR® No-ROX Kit (Bioline, London, UK) in a LightCycler® 480 System (Roche Molecular Systems, Inc., Pleasanton, CA, United States). The following PCR amplification protocol was used: 95 °C for 2 min, 40 cycles (95 °C for 5 s, 60 °C for 10 s, 72 °C for 20 s). The relative expression level of *SpDRM2* was calculated using the ΔΔCT method (Pfaffl, 2001) available in GenEx 6.0 (MultiD Analysis AB, Sweden). *SpTP41* (SapurV1A.0019s0010.1), *SpPPA2A3* (SapurV1A.0786s0040.1) and *SpPP2A1* (SapurV1A.0786s0040.1) transcripts were used as internal controls, as they were proven to be stably expressed among the tested conditions. For each gene, the average amplification efficiency was calculated based on fluorescence curves, using LinRegPCR software (Ruijter et al., 2009). Primer sequences used for RT-qPCR analysis and their efficiency are provided in Supplementary Table S2.3.

S2 - Supplementary Tables

Supplementary Table S2.1 – Mean efficiency of *in vitro* co-transformation of *Salix purpurea* clonal *in vitro* lines with *Agrobacterium rhizogenes*.

Genotype	Construct	Age (weeks)	Plant culture medium	Number of analyzed plants	Co-Transformation efficiency (%)*
**Experiment A – Optimization of the culture media and explant age**					
**PSP43**	PGWAY-0	2	MS ½ macro	30	66.67

**PSP43**	PGWAY-0	2	MS 30	60	83.33
**PSP43**	PGWAY-0	5	MS ½ macro	41	63.41
**PSP43**	PGWAY-0	5	MS 30	56	67.86
**Experiment B – Genotype response**					
**PSP43**	PGWAY-0	2	MS 30	79	86.08
**PSP33**	PGWAY-0	2	MS 30	76	98.68

* Co-transformed root efficiency calculated at 21 dpi (days post-inoculation) as the percentage of plants presenting at least one fluorescent root over the total number of infected plants.

Supplementary Table S2.2- List of primers used to clone the gene *SpDRM2*

Primer name	*Primer Sequence*
attB1_adapter	GGGGACAAGTTTGTACAAAAAAGCAGGCT
attB2_adapter	GGGGACCACTTTGTACAAGAAAGCTGGGT
DRM2_ins_cds_F	AAAAAGCAGGCTTAATGGTTGGTGATTCTTTATGTGGTGATAATG
DRM2_ins_cds_R	AGAAAGCTGGGTTTCAACTGTATCTAGATGTTAAATTCTTGACAACATCTAGG

Supplementary Table S2.3 – List of primers used in RT-qPCR performed in this study.

Gene name	*Transcript*	*Primer F*	*Primer R*	*Efficiency*
SpPP2A3	SapurV1A.0786s0040.1	ACGTTCTTGCTTCCCTTTCC	CAAGCTGTTTATCCGTTTCAAG	0.879
SpPP2A1	SapurV1A.0113s0310.1	CAGTGAAGAAATGGGCAAGG	CCAGATTGAGATGGCTGAAAA	0.890
SpTP41	SapurV1A.0019s0010.1	CGATCGAATCCGTATTAAAATTCC	CGGCTTTTAGGTCTTTGTCATCTAC	0.900
SpDRM2	SapurV1A.0571s0130.1	TTCGCTCATCGAGTCTGAGGCTTCC	ACAGATGAGCTTGCCTCGAGAGAG	0.728
